# A Systematic Review of Pharmacologic and Rehabilitative Treatment of Small Fiber Neuropathies

**DOI:** 10.3390/diagnostics10121022

**Published:** 2020-11-28

**Authors:** Michele Vecchio, Rita Chiaramonte, Marcello Romano, Piero Pavone, Giuseppe Musumeci, Giulia Letizia Mauro

**Affiliations:** 1 Department of Biomedical and Biotechnological Sciences, Section of Pharmacology, University of Catania, 95123 Catania, Italy; 2 Rehabilitation Unit, “AOU Policlinico Vittorio Emanuele”, 95123 Catania, Italy; 3 Neurology Unit, Azienda Ospedaliera Ospedali Riuniti Villa Sofia Cervello, 90146 Palermo, Italy; mc.romano1958@gmail.com; 4 Department of General Paediatrics A.O.U., Policlinico-Vittorio Emanuele University Hospital, 95123 Catania, Italy; ppavone@unict.it; 5 Department of Biomedical and Biotechnological Sciences, Anatomy, Histology and Movement Science Section, School of Medicine, University of Catania, 95123 Catania, Italy; g.musumeci@unict.it; 6 Research Center of Motor Activities (CRAM), University of Catania, 95123 Catania, Italy; 7 Department of Biology, College of Science and Technology, Temple University, Philadelphia, PA 19122, USA; 8 Department of Surgical and Oncology Sciences, University of Palermo, 90127 Palermo, Italy; giulia.letiziamauro@unipa.it

**Keywords:** small fiber neuropathy, treatment, systematic reviews

## Abstract

The aim of this systematic review is to guide the physician in defining the pharmacologic and rehabilitative therapeutic approaches for adopting the best strategies described in the current literature. The search was conducted in PubMed, EMBASE, Cochrane Library and Web of Science to identify the treatment of small fiber neuropathies. Two reviewers independently reviewed and came to a consensus on which articles met inclusion/exclusion criteria. The authors excluded the duplicates, animal studies and included the English articles in which the treatment of patients with small fiber neuropathies was described. The search identified a total of 975 articles with the keywords “small fiber neuropathy” AND “rehabilitation” OR “therapy” OR “treatment”. Seventy-eight selected full-text were analyzed by the reviewers. Forty-two publications met the inclusion criteria and were included in the systematic review to describe the rehabilitative and pharmacologic treatment of small fiber neuropathies. Despite the range of different protocols of treatment for small fiber neuropathy, other robust trials are needed. In addition, always different therapeutic approaches are used; a unique protocol could be important for the clinicians. More research is needed to build evidence for the best strategy and to delineate a definitive therapeutic protocol.

## 1. Introduction

### Small Fiber Neuropathy

Small fiber neuropathy (SFN) is caused by the impairment of unmyelinated C and thinly myelinated Aδ fibers. The symptoms are characterized by sensory symptoms, pain and autonomic symptoms, such as palpitations, gastrointestinal disturbances, and orthostatic dizziness. Neuropathic symptoms have a negative impact on the quality of life [1]. The symptoms and signs can be present as spontaneous (e.g., burning, deep, itching and paroxysmal) or evoked (e.g., thermal allodynia, light tough allodynia and hyperalgesia) pain.

Our systematic review defined the different rehabilitative and pharmacological approaches for SFN and to guide the physician to delineate a therapeutic protocol adopting the best strategies described in the current literature. In addition, we analyzed all the therapeutic approaches we found in the current literature to realize a guide to provide a common language to the multidisciplinary team such as physiatrists, neurologists, physiotherapists, nurses and neuropsychologists that must treat this disorder. The current literature did not describe a unique therapeutic approach, use arbitrarily different therapies. A therapeutic protocol should make more objective, reproducible, repeatable the outcomes and could help the multidisciplinary team to manage the patients.

## 2. Methods

### 2.1. Search Strategy

The search was carried out on the following medical electronic databases: PubMed, EMBASE, Cochrane Library and Scopus, Web of Science. The reference list of the related articles was also used to search for other suitable documents. The review was conducted from 22 May 2020 to 1 July 2020.

### 2.2. Selection Criteria and Data Extraction

Studies considered for this review must include the therapeutic methods in patients with SFN. We included English original articles about the rehabilitation and the pharmacological approaches for the SFN. We excluded animal studies, participants with other neuropathies. We also excluded all of the remaining duplicates.

Two reviewers (C.R. and V.M.) independently screened the titles and abstracts from the initial search to identify relevant records and to identify eligible studies based on title and abstract. Selected full texts were then reviewed and included in the systematic review, following the PRISMA protocol [2] and in accordance with the PICOS (population, intervention, comparison, outcome, and study design) criteria [3] shown in Table 1: Participants were all patients affected by SFN; intervention was based on rehabilitation therapy or pharmacological approaches; the comparator was any comparator; the outcomes included clinical assessments, diagnostic scales, electromyography and nerve conduction, biopsy; and study design was randomized clinical trials (RCTs), case series and case report, retrospective studies.

## 3. Results

### 3.1. Description of the Studies

**Figure 1:**
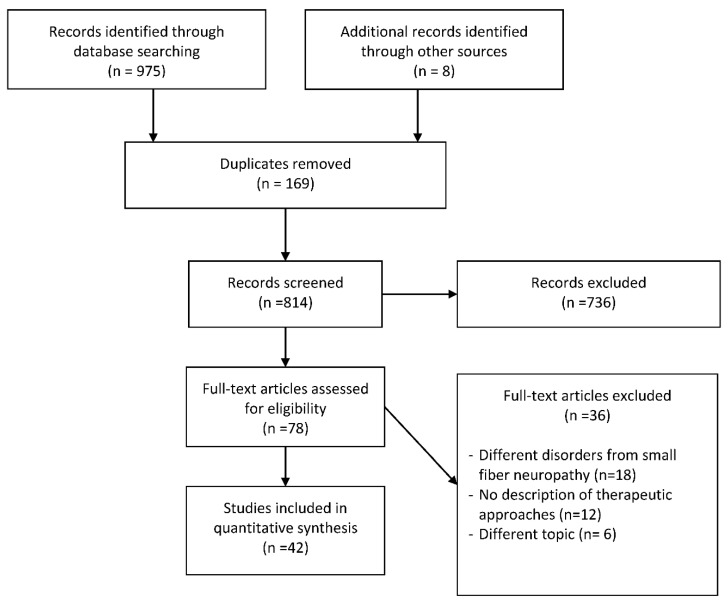
Flowchart of the process of literature search and extraction of studies meeting the inclusion criteria.

From 1984 to 2019, the database search of 975 articles with the following MeSH terms, words and combinations of words “small fiber neuropathy” AND “rehabilitation” OR “therapy” OR “treatment”, whose titles and abstracts were screened by the reviewers. The papers that remained for full-text screening were 78, and the eligibility of the study inclusion was assessed independently. Forty-two publications met the inclusion criteria and were included in the systematic review. Thirty-six were excluded for the following reasons: 18 involved individuals with different disorders from SFN, 6 examined different topics from our aim, 12 did not present any therapeutic procedure (Figure 1).

The qualitative information synthesis for each parameter was attributed to the following evidence levels according to the recommendations of the Oxford Center for Evidence-Based Medicine: evidence from a systematic review of randomized controlled trials (1a), controlled clinical studies (2a), case–control-studies (3a) and from non-systematic reviews (4) (Table 1).

### 3.2. Variations of Experimental Conditions across the Studies

The selected 42 articles were described on the basis of the several therapeutic methods used in each study for the treatment of SFN. Characteristics of the studies are shown in Table 1.

All study groups were not homogeneous for relevant general clinical features as clinical presentation, duration of disease and of the symptoms, kinds of diagnostic measures, the severity of symptoms, rehabilitation and pharmacological therapy, time of starting therapy, duration of treatment, the follow-up period at the end of the therapy (Table 1).

### 3.3. Pharmacologic and Rehabilitation Therapy

Many different treatments were experienced. Opioid analgesics [8,21,24] or nonopioid analgesic [22,26], corticosteroids [8,10,33,34,37,44], intravenous immunoglobulin (IVIG) alone [8,12,15,23,27,31,37,39,46,47] or in combination with other specific drugs, such as azathioprine [29], anti-epileptic drugs [4,11,13,16,18,28,32], immunotherapy [14,19,37], hormone therapy [7,43]. Less used are the following therapeutic strategies, in used for specific disorders, such as ARA290, an erythropoietin derivate for sarcoidosis SFN [45], recombinant human nerve growth factor for diabetic SFN [5], propranolol for SFN related to aquagenic pruritus [9], plasma exchange therapy for complex regional pain syndrome [6], enzyme replacement therapy for Fabry related SFN [17,35], botulinum toxin type A for keloid [38].

Furthermore, two specific surgery strategies were described: the stellate ganglion blockade for SFN causing burning mouth syndrome [41] and the dorsal root ganglion stimulation for neuropathic pain of feet [25]. 

Motor exercises and a rehabilitative program could be part of the treatment strategy [18,20,21,30,36,40,42].

## 4. Discussion

Our systematic review focused on the several pharmacological and rehabilitative therapies used for SFN. We realized a comprehensive overview to give a guide to ease the collaboration of a multidisciplinary team.

### 4.1. Comparing Studies: Therapeutic Strategies

To choose the correct therapeutic approach, the first step is to confirm the diagnosis. Then it is essential to search for associated conditions because these could be treatable [48]. 

Several causes of SFN are potentially treatable [49], such as metabolic syndrome [50,51], and type 2 diabetes [52] associated with SFN. If the condition is not preventable, pharmacologic treatment and rehabilitation could improve the impairment and the quality of life. The treatment of symptoms is mandatory, and the possibility to add exercises and rehabilitation programs could permit to avoid disability and to maintain an adequate quality of life [53].

### 4.2. The Pharmacological Approaches

The management of neuropathic pain has been a challenging task for physicians [24]. There is limited evidence on the effectiveness of specific medications for the treatment of pain associated with SFN, and the most commonly used medications include antidepressants, anticonvulsants, mexiletine, topical agents, opiates and neuromodulation [54,55]. The guidelines for the pharmacologic management of neuropathic pain and diabetic painful polyneuropathies of the American Academy of Neurology (AAN) and the European Federation of Neurological Societies (EFNS) [56,57] could be adopted for the treatment of SFN. The opioid analgesics may contribute to a centrally-sensitized pain state, which may be refractory to other symptomatic approaches [58], with the activation of microglial cells [58] and of the central glutaminergic system [59]. About 45% of sarcoidosis-related SFN was treated with opioid analgesic therapy as the first approach [8]. In the case report of Mishra et al. [26], flupirtine, a nonopioid analgesic with muscle-relaxing properties, reduced neuropathic pain. Keohane et al. [22] proposed tafamidis, a non-NSAID highly specific transthyretin stabilizer, to delay the neurologic disease progression in the early-stage of transthyretin V30M familial amyloid polyneuropathy. A neuropathic pain related to SFN secondary to a keloid was treated successfully with botulinum toxin type A [38].

Immunotherapy with infliximab [19,37] or adalimumab [14] could play a crucial role in modifying the pathogenesis of SFN in immune-mediated inflammatory diseases [19]. 

The use of corticosteroids, immunosuppressive and anti-epileptic drugs showed discordant results. No improvements were reported in neuropathic symptoms and pain intensity after corticosteroid treatment in Sjogren, sarcoidosis and Guillain–Barré related SFN [8,37,44], or marked clinical improvement, according to other studies [10,33,34]. No clinical improvements were noted with methotrexate [37], but positive results with mycophenolate mofetil [33]. Nevoret et al. [29] added azathioprine to IVIG therapy, with consequent improvement in neuropathic symptomatology. 

The benefits of intravenous immunoglobulin (IVIG) were reported for neuropathic pain in Sjogren [8,27]. In 8 studies, the efficacy and safety of IVIg are evaluated in patients with different features of SFN [12,15,23,27,31,37,39,46,47]. In contrast, IVIG had disappointing results, according to Pereira et al. [33] and Yuki et al. [44].

Gonzalez-Duarte et al. [16] showed improvements in prediabetic neuropathic pain after pregabalin treatment. De Greef et al. [11,13], Namer et al. [28] and Brouwer et al. [60] assessed the efficacy, safety, and tolerability of lacosamide, an anticonvulsant, in patients with SCN-associated small fiber neuropathy. Carbamazepine is useful to reduce SFN-related neuropathy [32] too. Gabapentin and naproxen [4] or duloxetine [18] were used for SFN associated with hantavirus infection [4] or in the absence of results with other therapies [4].

Enzyme replacement therapy (ERT) with recombinant human α-galactosidase significantly improved the function of C-, AΔ-, and Aß- nerve fibers and intradermal vibration receptors in Fabry neuropathy [17]. But according to Schiffmann et al. [35] epidermal nerve fiber regeneration did not occur after ERT. Van Velzen et al. [45] described the long-lasting beneficial effects of ARA290, an erythropoietin derivate, in symptoms of sarcoidosis-related SFN in patients. According to Apfel et al. [5], recombinant human nerve growth factors had significant beneficial effects on diabetic polyneuropathy. In the case report of Cao et al. [9], SFN related to aquagenic pruritus was treated with propranolol with significative benefit after a month of therapy. Plasma exchange therapy is effective in patients with severe long-standing complex regional pain syndrome [6].

### 4.3. Surgical Approaches

Stellate ganglion blockade [41] for recalcitrant pain in burning mouth syndrome and dorsal root ganglion stimulation [25] induced paraesthesia covering the entire pain area could be an effective therapy in SFN.

### 4.4. Rehabilitative Program

Another important field of therapy in SFN is the rehabilitation that could be added at a pharmacologic treatment [21,36] or used in the absence of pharmacologic results [18] and could be the first step of a therapy protocol (Table 2).

### 4.5. Implication in Rehabilitation

Early recognition of SFN is important to start an appropriate and prompt treatment.

The aim of therapy is to relieve the neuropathic symptoms. The reduction of pain and the improvement in quality of life and in the ability to participate in activities is the purpose of rehabilitation approaches and could be the best complementary treatment to pharmacologic strategies. Specific exercises with proprioceptive and superficial sensibility stimulation could enhance recovery. Exercise may positively influence the pathological factors associated with neuropathy by promoting microvascular dilation, reducing oxidative stress, and increasing neurotrophic factors [61,62].

## 5. Limitations

A lack of uniformity among the papers (measured parameters and assessment scale) may affect the outcomes of considered articles. The absence of information about some clinical characteristics that could influence the symptomatology represents another limitation, such as comorbidities, the use of other specific drugs, psychologic traits. Furthermore, in some articles, the sample was very small. Several studies did not assess participants’ educational status; it could be a confounding factor and could influence the results.

## 6. Conclusions

The treatment of SFN is indispensable for the improvement of quality of life of individuals with neuropathic symptoms. SFN has a negative psychosocial impact on the lives of the patients and of their families.

We showed all the therapeutic approaches described in the current literature for SFN. On the basis of the different treatments, the physicians could obtain a guide and a common protocol for a multidisciplinary team. Despite the range of therapies for SFN, robust trials miss and always different therapeutic approaches are used. A comprehensive overview could give a guide to the physicians, and a complete protocol could ease the therapeutic and diagnostic approach to small fiber neuropathies. More research is needed to build evidence for the best therapy and to delineate a definitive therapeutic protocol.

## Figures and Tables

**Table 1:** Treatment of SFN. Characteristics and outcomes of studies included in the systematic review.

Authors, Year	Study Design	Patients. Age	SFN Disease. Age at Onset Diagnosis	Onset SFN Symptoms	Symptoms	Therapy	Conclusions
Anderson 2017 [4]	Case report	1 patient, 35 years old	SFN associated with hantavirus infection	One month after hantavirus infection	Severe, intractable burning limb pain. Allodynia to light touch and hyperalgesia to pinprick in a stocking distribution up to the mid-calf bilaterally	Gabapentin and naproxen	At follow-up 4 months later, his limb pain was only marginally improved
Apfel 2000 [5]	Clinical trial level 2	A: 418 rhNGF B: 461 placebo 18–74 years	Diabetic SFN	-	Neuropathic pain	rhNGF	Significant beneficial effect of rhNGF on diabetic polyneuropathy
Aradillas 2015 [6]	Case series level 4	33 p, 45.7 years	SFN related to complex regional pain syndrome	9.7 years	Neuropathic pain	Plasma exchange	Plasma exchange is effective for patients with severe long-standing complex regional pain syndrome
Azmi 2015 [7]	Observational study level 2	49 patients A: 18 patients with subcutaneous insulin infusion 55.4 ± 2.9 years B: 31 patients with daily insulin injection 49.9 ± 3.3 years	Diabetic SFN	A: 34.8 ± 3.1 years B: 35.2 ± 3.6 years	Neuropathic pain	Continuous subcutaneous insulin Infusion	Daily insulin injection group showed no significant change, but the subcutaneous insulin infusion group showed an improvement in corneal nerve morphology, consistent with regeneration
Birnbaum 2018 [8]	Observational study level 2	23 patients ~53.6 years 44 ± 13 years	Sjögren’s syndrome	49.5 ± 23 years	Pain. Eleven patients had stocking-and-glove pain, and 12 patients had non-stocking and-glove pain. Ten SFN patients (~45%) had neuropathic pain preceding sicca symptoms.	Opioid analgesics were prescribed to ~45% of SFN patients	Sjögren’s syndrome SFN had increased frequency of male sex, decreased frequency of multiple antibodies, were frequently treated with opioid analgesics, and could present with nonstocking-and-glove pain
Cao 2015 [9]	Case report	1 patient 36 years	SFN related to idiopathic aquagenic pruritus	~for 3 y after symptoms	Aquagenic pruritus	Propranolol 10 mg bis in die for 1 month	Atenolol is to be preferred to propranolol, in view of its convenient once-a-day dosing and better side effect profile
Dabby 2006 [10]	Observational study level 2	4 patients ~49 years	Idiopathic SFN	-	Neuropathic pain. Symptoms were distal and symmetrical in three patients and generalized in one patient	Prednisone, 1 mg/kg for 12 weeks	Clinical improvement occurred 1–2 weeks after oral prednisone therapy was initiated.
De Greef 2016 [11]	Clinical trial level 2	25 patients 18–80 years	SCN9A-associated SFN	-	Pain, altered temperature sensation.	Lacosamide, 200 mg bis in die for 8 weeks	Lacosamide: a potential treatment option in patients with painful neuropathies, considering the central role of Nav1.7 in pain.
De Greef 2016 [12]	Clinical trial level 2	60 patients >18 years	Idiopathic SFN	-	Pain, altered temperature sensation.	Intravenous Immunoglobulins g/kg body weight over 2–4 consecutive days, followed by a maintenance dose of 1 g/kg body weight over 1–2 consecutive days given 3 times at a 3-weeks interval	Positive findings in SFN after intravenous immunoglobulins
De Greef 2019 [13]	Clinical trial level 2	24 patients ~48 years	SCN-SFN	-	Pain and autonomic dysfunction	Lacosamide, 200 mg bis in die for 8 weeks	Significant effect on pain, general wellbeing, and sleep quality
Favoni 2018 [14]	Case report	1 patient 45 years	Anti-GQ1b antibodies associated with SFN	~2 y after symptoms	Tingling and burning pain sensation in the arms and legs, with nocturnal exacerbation	Adalimumab: 40 mg every day, subcutaneous administration for 1 year	Benefit from immunotherapy
Gaillet 2019 [15]	Retrospective study level 2	11 patients 41–62 years	Sjögren’s syndrome	~6.5 y after symptoms	Pain	6 months intravenous immunoglobulins infusions, 0.4 g/kg/day for 5 days	Efficacy of intravenous immunoglobulins treatment for pain relief in Sjögren’s Syndrome-SFN with an improvement of quality of life and sensory testing
González-Duarte 2015 [16]	Clinical trial level 2	45 patients ~54 years	Prediabetic SFN	-	Neuropathic pain	Pregabalin was initiated at a dose of 75 mg and tapered up to 300 mg bis in die.	Improvement of prediabetic neuropathic pain with pregabalin
Hilz 2004 [17]	Observational study level 2	22 patients A: 11 patients B: 11 patients 27.9 ± 8 years C: 25 HC 29 ± 10.4 years	Fabry related SFN	-	Pain	Enzyme replacement therapy A: for 18 months B: for 23 months C: placebo Every 2 weeks, patients were treated with 0.9 to 1.1 mg/kg of agalsidase ß	Enzyme replacement therapy with agalsidase beta significantly improves function of C-, AΔ-, and Aß- nerve fibers and intradermal vibration receptors in Fabry neuropathy
Hoeijmakers 2016 [18]	Case reports	2 patients ~15 years	1 p idiopathic SFN, 1 p diabetic SFN	~7 y after symptoms	Painful itch and tingling of legs, dysautonomia symptoms	Gabapentin	Moderate pain relief with treatment with gabapentin in a case. Treatment with duloxetine, combined with a rehabilitation program, resulted in a marked improvement in daily functioning.
Hoitsma 2006 [19]	Observational study level 2	1 patient 39 years	Sarcoidosis-associated SFN	-	fatigue, neuropathic pain, autonomic dysfunction, and arthralgia	Infliximab	SFN seems not an irreversible disorder; infliximab had good outcomes
Hong 2013 [20]	Case report	1 patient 64 years	Diabetic SFN	~2 years	Peripheral neuropathic pain in his both feet	Vibration therapy 3 min of vibration Treatment (total 12 min) at 20 Hz 5 times a week for 4 weeks	The whole-body vibration is a good complementary treatment
Kluding 2012 [21]	Observational study level 2	17 patients 58.4 ± 5.98 years	Diabetic SFN	12.4 ± 12.2 years	Pain	10 weeks aerobic and strengthening exercises	Exercises improve SFN symptoms
Keohane 2017 [22]	Clinical trial level 1	A: 48 patients B: 44 patients 18–75 years	Amyloid SNF	-	Distal-to-proximal sensorimotor neuropathy with autonomic symptoms	A: Tafamidis, 20 mg/d for 18 months B: placebo	Tafamidis delays neurologic progression in early-stage ATTRV30M-FAP.
Liu 2018 [23]	Retrospective study level 2	55 patients 41 ± 17 years	Autoimmune SFN	6.3 ± 6.3 years	Neuropathic pain	Intravenous immunoglobulins ≥1 g/kg/4 weeks for ≥3 months.	Intravenous immunoglobulins are safe and effective
MacDonald 2019 [24]	Retrospective study level 2	87 patients	SFN	3.2 years	Neuropathic pain	Gabapentin (*n* = 69), pregabalin (*n* = 51), duloxetine (*n* = 41), tricyclic agents (*n* = 37), and topical cream (e.g., capsaicin, lidocaine; *n* = 29).	45.5% of patients had, at some time, been treated with opioid medications for neuropathic pain.
Maino 2017 [25]	Case report	1 patient 74 years	SFN	~6 years after symptoms	Burning and shooting pain in feet	Dorsal Root Ganglion Stimulation	20 months post-implantation, the patient continued to experience stimulation-induced paresthesia covering the entire pain area
Mishra 2012 [26]	Case report	1 patient 22 years	SFN	~6 months after symptoms	Neuropathic pain	Flupirtine 200 mg 3/d along with pregabalin 300 mg 2/d	Reduction of pain after flupirtine
Morozumi 2008 [27]	Observational study level 2	5 patients 61.8 years	Sarcoidosis-associated SFN	-	Neuropathic pain	Intravenous immunoglobulins 0.4 g/kg/d for 5 days	Beneficial after intravenous immunoglobulins therapy
Namer 2019 [28]	Case report	1 patient 69 years	SNF	~10 years after symptoms	Burning pain	Lacosamide 50 mg orally in the evening for 6 months	Lacosamide reduced pain in SFN
Nevoret 2014 [29]	Case report	1 patient 60 years	Chronic inflammatory demyelinating polyneuropathy SNP	~2 years	Neuropathic pain	Intravenous immunoglobulins: 6 doses total, 75 g each + Azathioprine 50 mg bis in die	Less burning, shooting pains and tingling
Otis 2013 [30]	Clinical trial level 2	20 patients 62.5 ± 10.9 year	Diabetic SFN	-	Neuropathic pain	A: 12 p cognitive-behavioral therapy B: 8 p traditional treatment	Cognitive-behavioral therapy reduced pain
Parambil 2010 [31]	Case series	3 patients	Sarcoidosis-associated SFN		Intractable neuropathic pain, autonomic dysfunction	Intravenous immunoglobulins: 2-g/kg followed by 1-g/kg in 2-weeks, and then received maintenance doses of 1-g/kg every 4-weeks.	Intravenous immunoglobulins appear to be effective in relieving symptoms
Patel 2019 [32]	Case report	1 patient 31 years	SCN-SNF	~10 years after symptoms	Erythromelalgia, painful flushing and burning paresthesia of the proximal extremities	Carbamazepine 200 mg bis in die	Carbamazepine reduced pain
Pereira 2016 [33]	Case series	13 patients 55 years	Sjögren’s syndrome	~3 years after symptoms	Neuropathic pain, Paresthesia	7 p corticosteroids, 7 p mycophenolate mofetil, 6patients hydroxychloroquine, 5 patients intravenous immunoglobulins, 4 patients cyclophosphamide, 2 patients other immunosuppressive drugs	Treatment with corticosteroids with immunosuppressive drugs, as mycophenolate mofetil, had positive results. In contrast, intravenous immunoglobulins had disappointing results
Saito 2015 [34]	Case report	1 patient 59 years	Sarcoidosis-associated SFN	10 days	Progressive pain and hypoesthesia of the right lower back associated with fever and constipation	Methylprednisolone 1 g/d for 3 days, followed by prednisolone 40 mg/d	Neurological symptoms were effectively relieved with high-dose steroid therapy
Schiffmann 2006 [35]	Clinical trial level 2	25 patients ~34 years	Fabry disease-related SFN	-	Neuropathic pain	α galactosidase 0.2 mg/kg every 2 weeks followed for 12 months	Epidermal nerve fiber regeneration did not occur after enzyme replacement therapy
Smith 2006 [36]	Observational study level 2	32 patients 60 ± 8.4 years	Diabetic SFN	7 ± 31 years	Neuropathic pain	Rehabilitative exercises	Rehabilitative exercises improved symptoms
Tavee 2016 [37]	Retrospective study level 2	- 115 patients - ~46 years, - 62 patients IVIG, - 12 patients infliximab - 14 p IVIG + infliximab - 27 patients not treated	Sarcoidosis-associated SFN	41 years	Pain, paresthesia, dysautonomic symptoms	Intravenous immunoglobulins 2 mg/kg bodyweight for 5 days; Anti-TNF alpha (infliximab) 5 mg/kg every 4 weeks	Beneficial from intravenous immunoglobulins and anti-TNF alpha in symptoms
Uyesugi 2010 [38]	Case report	1 patient, 80 years	Keloid related SFN	5 years after surgery	Itching, pain, and allodynia	Botulinum toxin type A, 100 U, diluted with 5 mL of preservative-free saline	A keloid was treated successfully with botulinum toxin type A.
Wakasugi 2009 [39]	Case report	1 patient, 40 years	Sarcoidosis-associated SFN	2 months	Paresthesia and burning pain in the distal upper and lower limbs.	Intravenous immunoglobulins 2 mg for 5	Intravenous immunoglobulins therapy was immediately and extremely effective
Waldinger 2011 [40]	Case reports	2 patients ~54.5 years	SFN	~2.5 years	Unpleasant genital sensations of being on the edge of an orgasm, overactive bladder, absence of erection and ejaculation, or spontaneous ejaculations	TENS	In the male patient, the use of TENS clinically significantly reduced the symptoms of restless genital syndrome, in a female patient, TENS application had no effect on genital complaints and complaints of overactive bladder syndrome
Walega 2014 [41]	Case report	1 patient 53 years	Burning mouth syndrome-related SFN	~6.5 months	Bilateral burning pain in the anterior tongue and mucosa of the lips	Verbal rating scale, Patient’s Global Impression of Change	Bilateral stellate ganglion blockade
Weintraub 2009 [42]	Clinical trial level 1	A: 90 patients 61.1 ± 10.4 B: 104 patients 60.6 ± 12.4 years	Diabetic SNF	-	Neuropathic pain	A: pulsed electromagnetic fields varying intensity and polarity 10–30 min session for 2 h maximum, daily, 12 w B: Sham group	Pulsed electromagnetic fields at this dosimetry were ineffective in reducing neuropathic pain
Windebank 2004 [43]	Clinical trial level 2	A: 20 patients, 58.3 ± 12.2 years B: 20 patients 62.2 ± 10.7 years	SFN	>6 months	Painful, distal, symmetrical neuropathy	A: IGF-I, 0.05 mg/kg twice daily for 6 months B: placebo	IGF-I was safe but did not improve symptoms in this 6-month trial
Yuki 2018 [44]	Case report	3 patients, ~27.3 years	SFN variant of Guillain–Barré syndrome	The three patients developed the symptoms 42, 6 and 11 d, respectively, after symptom onset	Pinprick sensation with hyperesthesia and brush allodynia in a glove-and-stocking distribution	1 p oral prednisolone 40 Mg/d for 5 days 2 patients intravenous immunoglobulins	One patient showed no response to intravenous immunoglobulins but a good response to prednisolone. One patient had no significant improvement with prednisolone. One patient had a gradual spontaneous recovery
van Velzen 2014 [45]	Clinical trial level 2	A: 12 patients B: 13 patients ~48.6 years	Sarcoidosis-associated SFN	7 years between the current study and the diagnosis of sarcoidosis	Pain, allodynia, hyperalgesia	A: ARA290, an erythropoietin derivate, intravenous of 2 mg dissolved in 6 mL of normal saline, 3/weeks for 4 weeks B: placebo	Long-lasting beneficial effects of ARA 290

Small-fiber neuropathies (SFN), rhNGF recombinant human nerve growth factor, insulin-like growth factor-I (IGF-I), Transcutaneous electrical nerve stimulation (TENS).

**Table 2:** Rehabilitation programs.

Authors	Rehabilitation Program	Note
Hoeijmakes 2016 [18]	-	After no results with a pharmacologic approach, the rehabilitation program was added
Hong 2013 [20]	Four bouts of 3 min of vibration treatment (total 12 min) at 20 Hz five times a week for four weeks.	Body vibration reduced acute and long-term pain in diabetics
Kluding 2012 [21]	10-week exercise program with both aerobic and strengthening and resistance exercises significantly improved selected measures of peripheral nerve function, with a reduction in pain and neuropathic symptoms	The rehabilitation improved the neuropathic symptoms, nerve function and cutaneous innervation Rehabilitative exercises were added to the pharmacologic approach in diabetic SFN
Otis 2013 [30]	Cognitive behavioral: each session of 60 min for 11 weeks	For neuropathic pain in diabetic neuropathy
Smith 2006 [36]	80 min for 73 weeks with improvement in neuropathic symptoms	Rehabilitative program was added to the pharmacologic approach in diabetic SFN
Waldinger 2011 [40]	Transcutaneous electrical nerve stimulation (TENS) in SFN for restless genital syndrome (ReGS) of dorsal nerve penis and in overactive bladder syndrome (OAB).	-
Weintraub 2009 [42]	Pulsed electromagnetic fields was ineffective in reducing diabetic neuropathic pain	-
